# How creative destruction functions in corporate entrepreneurial process: an empirical investigation of Schumpeterian concept in engineering firm settings in Pakistan

**DOI:** 10.1186/s13731-022-00199-3

**Published:** 2022-01-25

**Authors:** Muhammad Zubair Alam, Shazia Kousar, Muhammad Rizwan Ullah, Amber Pervaiz

**Affiliations:** 1 Department of Management Science, Superior University, Lahore, Pakistan; 2 Lahore College for Women University, Lahore, Pakistan; 3 Lyallpur Business School, Government College University, Faisalabad, Pakistan; 4 Department of Economics, University of Education, Lahore, Pakistan

**Keywords:** Creative destruction, Market orientation, Technical opportunity recognition, Corporate entrepreneurship, Schumpeter's entrepreneur

## Abstract

Schumpeter's idea of creative destruction (CD) explains innovation functions in organisations. This paper investigates the CD concept in engineering firms by explaining how technical opportunity (TO) transforms into corporate entrepreneurship (CE) actions once opportunities have a market orientation (MO). A **s**urvey was conducted using a structured questionnaire with 132 managers of engineering firms in Pakistan. Structural Equation Modelling (SEM) using Partial Least Square (PLS) approach has been used to analyse the data. Results reveal that MO and TO exerts a positive influence on CE. MO is the reason for the emergence of TO, which corporate entrepreneurs in engineering firms exploit. CD intensifies the impact of MO on TO significantly. Opportunity recognition in engineering firms is distinguished and bounded by MO and technical viability. Engineering firms need to identify gaps in the market through naturally occurring obsolescence of products and services (CD) to create TO with appropriate MO. This study has revived a classical debate over opportunity recognition by incorporating external factors to propose the CE model. The Schumpeterian opportunity recognition process and CD have been examined for engineering firms.

## Introduction

History has proved that businesses should be capable of rapid change, transformation, and innovation to fulfil the fast-changing marketplace's expectations (Schoemaker et al., 2018). In this regard, corporations should improve their innovation capability (Kelley et al., 2009) through CE (Lee & Pati, 2017). Various researchers also indicated entrepreneurship (ENTR) as the fundamental act supporting innovation (Amit et al., 1993). Innovation reverberates with Schumpeter's (1934) view, i.e. "*ENTR is the primary catalyst for innovation*." This view is entirely concerned with entrepreneurial action as a crucial transformation mechanism.

Schumpeter (1934) believed that innovations are the centre of economic change as they trigger the gales of "*CD*". Schumpeter (1934) defined CD as a process of industrial transformation where new opportunities are introduced to the market at the cost of existing ones. CE is a process through which an organisation is enthusiastic to abandon routine to seek new opportunities (Zampetakis & Moustakis, 2010). Likewise, CE is liable for promoting the gales of CD within an organisation through inspection of new opportunities, resource acquisition, implementation, commercialisation, exploitation, and development of new products and services (Guth & Ginsberg, 1990).

Like uncertain influences or external jolts, various contextual factors impact an individual's and firm's motivation to pursue new opportunities. For example, in pandemic COVID-19, individuals and firms seek opportunities in different market-relevant emerging requirements. Krueger (1998) emphasised the global business environment and its threats as an entrepreneurial opportunity process. Environmental hazards and uncertainties are considered to emerge as a result of CD due to rapid technological changes in the market. As an outcome of engineering firms, the TO recognition process emerged in industrial products and services due to ever and continually changing technology resulting in CD.

Schumpeter (1934) believed that an entrepreneur requires TO for the development of new products. TO, technical skills, and distinctive technical capabilities positively affect the CE as it is the belvedere of knowledge that enables the up-gradation of the existing system or the development of the new system (Zahra et al., 1999). It is considered that TO usually arises from MO, which refers to the endless search for opportunities. Any opportunity which occurs in an organisation or market gives the path of exploitation by someone. It is an approach to business that identifies the customer's needs and tries to develop the products according to their needs. MO is also perceived as a basis of innovation and competitive advantage (Barrett & Weinstein, 2015).

Schumpeter (1934) advocates the emergence of entrepreneurial opportunity as a result of technological innovation. Sarasvathy et al. (2010) emphasised this opportunity recognition in known technological change under uncertain market demand. Also, a higher degree of technical knowledge about change influences TO recognition. MO will influence technical knowledge to pursue TO. An important question arises, "*Do engineering firms differ in opportunity identification approach*?".

Academic researchers have worked on the relationship between MO and CE (Renko et al., 2009; Sciascia et al., 2006) and found a strong relationship between entrepreneurial opportunity and CE (García-Morales et al., 2014). Still, the area of CE concerning TO is under discussion in academia, specifically in engineering firms. Therefore, the present study shifts the focus from the extensively researched area, i.e. the entrepreneurial opportunity, to the less focused area, i.e. TO, because employees in entrepreneurial engineering firms are always looking for a TO. In ENTR literature, the relationship between TO and CE lacks empirical evidence (Kim, 2018). The indirect link between MO and CE (through TO) also lacks elucidation in extant literature.

**Fig. 1:**
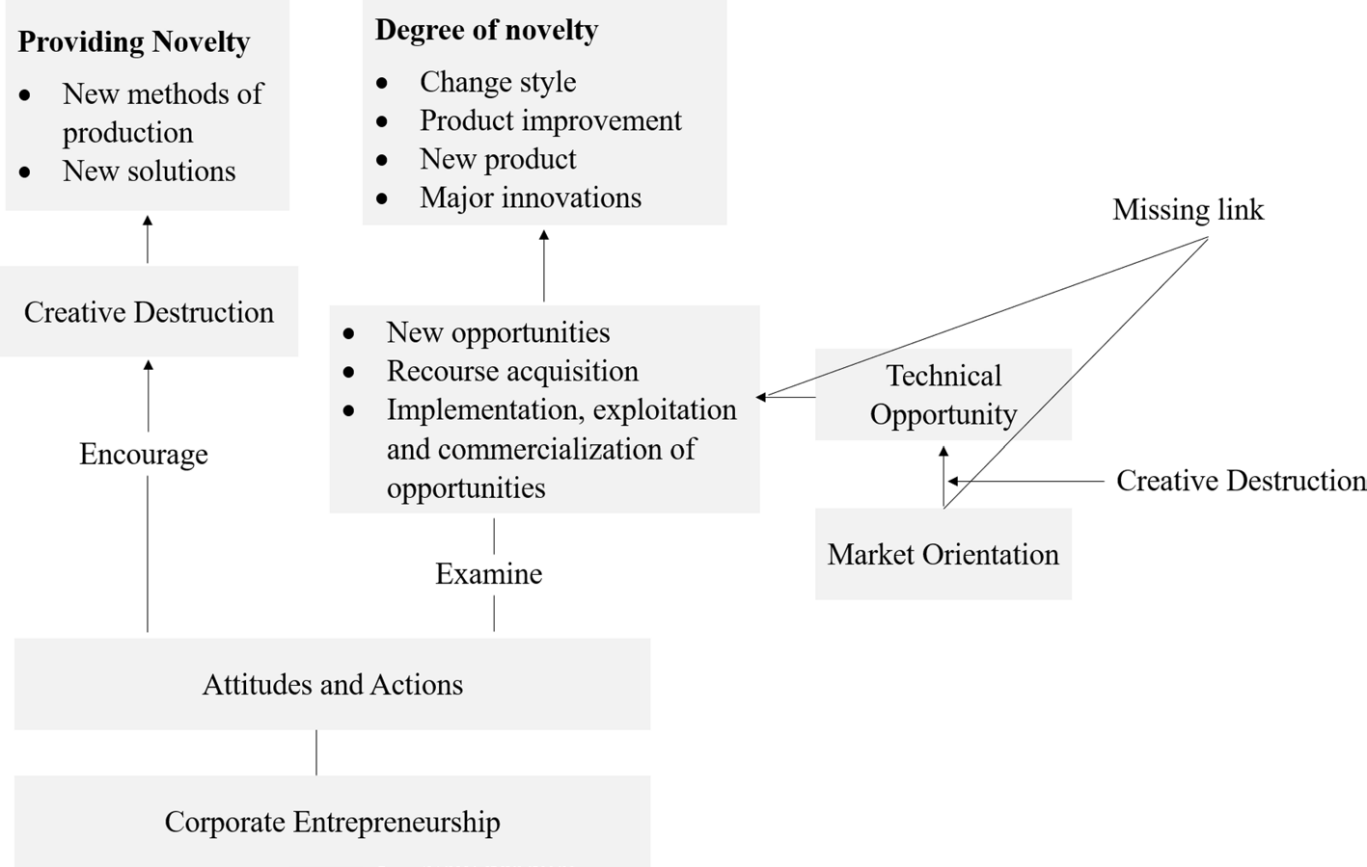
The missing link between TO, MO, CD, and CE

Furthermore, the moderating role of CD between MO and TO is to be considered as the same has not been empirically tested in engineering firms. Therefore, the present study contributes to the current debate of CE by empirically examining the missing link between TO, MO, CD, and CE in the context of engineering firms in Pakistan (shown in Fig. 1).

The present study significantly enhances the existing literature on CE in the engineering industry in Pakistan. The industry in Pakistan has progressed since 1947 (after independence from British Colonial rule), yet it is facing internal and external issues including availability of skills, lack of value addition, high cost of input, lack of competitiveness, issues of inward-looking, and low volume (Pakistan Business Council, n.d.). Textile being the leading industrial setup in Pakistan, other major industries include sugar, tobacco, food processing, cement, fertiliser, steel, chemicals, machinery and edible oil. Various issues, including MO and the TO identification, hinder Pakistan's industry from capturing a high share in the global market, resulting in higher imports and lower exports. This study offers a new approach for firms in the opportunity recognition process, which transforms from the inner organisational function of change (innovation) to look for gradual obsolescence of products and services in the market through the natural process of CD in the opportunity creation process.

## Literature review and hypothesis development

### Schumpeter's theory of innovation and corporate entrepreneurship

Joseph Alois Schumpeter is among the most significant economists who took part in the economic debate. His theory of innovation is the most distinctive contribution to economics (Hanusch & Pyka, 2007). According to Schumpeter, innovations are the fundamental factor of economic competitiveness and are the centre of economic change as they trigger the gales of "*CD*" (Schumpeter, 1934). Schumpeter defined CD as a process of industrial transformation, altering the economic structure from inside, i.e. through constant destruction of the old one and creating a new one. Schumpeter believed that "*entrepreneur*" plays an essential role in the process of innovation as they make the possible implementation of new combinations in business in the form of new products, new markets, and new methods of production.

The purpose of entrepreneurs is to transform the production process by exploiting the creation and opening of new sources of raw materials, or a new aperture for products, by rearranging a business. For this, the TO is considered an essential factor. By considering Schumpeter's view of innovation, i.e. implementing new combinations is an entrepreneurial activity, numerous researchers have observed that innovations play a crucial role in CE (Ireland et al., 2009; Schumpeter & Backhaus, 2003). His theory provides a clear theoretical justification for the relationship between TO, CD, and CE.

Schumpeter (1934) defined that entrepreneurs are the ones who carry out the new combinations in a business. He stated that "*the entrepreneurial function is the vehicle of a continual reorganisation of the economic system*" (pp. 155–156). The initial step to explain CE is to define those aspects of ENTR that explicate to the CE (Shin, 2013). Covin and Slevin (1991) stated that CE depends on product modification, risk-taking tendency, and dedication. Jennings and Lumpkin (1989) defined CE as the extent to which new products are developed. CE comprises two phenomena: the development of new products within existing organisations and the organisation's restoration through new ideas (Guth & Ginsberg, 1990). It is perceived as an organisational process that transforms individual thoughts into corporate action (Chung & Gibbons, 1997).

### Entrepreneurial and technical opportunity

Opportunity recognition is defined as: "*the process by which entrepreneurs see something that has the potential value*" (Ardichvili et al., 2003). Schumpeterian (creation) and Kirznerian (discovery) views are dialectics that are relevant to TO in engineering firms. Few researchers (e.g., Blaug, 2000) describe these views as complementary rather than opposing, but many scholars debated these views as opposing (Buenstorf, 2007). Kirzner (1997) advocates access to existing information, while Schumpeter (1934) stressed acquiring new knowledge in the market to recognise the entrepreneurial opportunity. The Discovery view of entrepreneurial opportunity recognition assumes that individuals and firms discover opportunities by recognising the value of new information instead of searching (Kirzner, 1997). The creation view takes the possibility of innovative products and services by creating innovative opportunities through search (Schumpeter, 1934).

Internal factors of an organisation impact entrepreneurial opportunity recognition; various research studies have stressed the importance of external environmental factors (Singh, 2000). Accordingly, consumer economics, political action, social values, technology, and regulatory standards instigate firms and individuals to pursue the opportunity. Shane (2003) and Schumpeter (1934) have viewed entrepreneurial opportunity emergence as a result of social and demographic changes, political and regulatory changes, and technological changes.

### Market orientation and corporate entrepreneurship

Corporate entrepreneurs create an environment that instigates innovative capacity (Antoncic & Hisrich, 2001). The linkage between CE and MO is perceived as the centre of business innovation and competitiveness (Barrett & Weinstein, 2015). MO refers to the continuous search for market opportunities and consistent reaction strategies that facilitate the firms to improve their performance (Im & Workman, 2004). MO occurs through the sequential process of intelligence generation, intelligence dissemination, and responsiveness of the firms (Kohli & Jaworski, 1990). MO is an arrangement of openness, and receptiveness of market intelligence (Kohli & Jaworski, 1990), which implies exploiting inventive things in response to market conditions (Slater & Narver, 1994). An efficient MO specifies a conviction to adjust to customers' unpredictable future needs (Atuahene‐Gima et al., 2005) and thus improve the conservation of competitive strategies and initiates innovative inducements (Zachary et al., 2011).

CE retains the business's strategic renewal, thus assuring its innovation and profitability (Drucker, 2007). Enterprises have to participate in entrepreneurial action to work efficiently in competitive markets (Zimmerman, 2010). The linkage between CE and MO is perceived as the centre of business innovation and competitiveness (Barrett & Weinstein, 2015). Considering the importance of MO, businesses researchers have started investigating the empirical relationship between MO and CE. For instance, the study of Barrett and Weinstein (2015) has shown a significant association between MO and CE as it provides the basis of innovation and competitive advantage. González‐Benito et al. (2009) revealed a positive relationship between MO and CE. Sciascia et al. (2006) indicated MO as an essential determinant of CE. A business should involve similar practical approaches to succeed in this vigorous era (Renko et al., 2009). The continuous search for market acquaintance demands maintaining an additional entrepreneurial alignment (Bojica et al., 2011). Therefore, the present study hypothesises that:

#### H1:

MO relates significantly and positively to CE.

### Technical opportunity, market orientation, and corporate entrepreneurship

Hansen et al. (2011) defined the opportunity as the possibility of introducing new products. An entrepreneur is always searching for an opportunity to develop new products or upgrade existing products. The current literature on entrepreneurship has specified that the achievement of entrepreneurial opportunity can be divided into opportunity, identification, and exploitation (Shane & Venkataraman, 2000).

Different views (e.g., cultural-cognitive and economic) emerged to explain the entrepreneurial opportunity identification process. The economic view describes that opportunity exists in the environment as an objective phenomenon (Companys & McMullen, 2007). As a result of this view, entrepreneurs are likely to recognise opportunities as a result of better prior knowledge (Shane & Venkataraman, 2000), being more alert (Gaglio & Katz, 2001) and with better information (Shane & Venkataraman, 2000). These antecedents of opportunity recognition are helpful after the gradual learning process. The cultural-cognitive view describes the entrepreneurial opportunity as a subjective phenomenon in which individuals create opportunity. Accordingly, the entrepreneurial opportunity exists when created and recognised by firms or individuals (Companys & McMullen, 2007, p. 305).

The present study considers entrepreneurial opportunity emanation from the MO. Any opportunity which arises in an organisation or market gives the path of exploitation by someone. It is an approach to business that identifies the customer's needs and tries to develop the products according to their needs. Schumpeter and Backhaus (2003) believed that an entrepreneur requires TO to create new products for which technical change is needed. According to Bhide (2003), about half of the founders of private companies (fortune 500) in the US indicated that change in technology or external factors was the reason for business initiation. Also, the increase in the technological change rate has resulted in a rise in entrepreneurial start-ups (Blau, 1987).

The present study shifts focus from the entrepreneurial opportunity to TO as corporate entrepreneurs in engineering firms always look for a TO. The concept of TO is drawn from Schumpeter's theory of innovation. Schumpeter specified that opportunity requires the development of new knowledge that comes from technological change. García-Morales et al. (2014) empirically tested the relationship between technological change and CE and showed that technological change tends to increase CE. Therefore, the present study proposes that:

#### H2:

TO relates significantly and positively to CE.

#### H3:

MO relates significantly and positively to TO.

### The mediating role of technical opportunity

For individuals, why, when, and how people discover opportunities is adequately addressed in research (e.g., Shane & Venkataraman, 2000), but opportunity recognition literature is scarce, especially for firms dealing with some engineering-related products or services. The present study contends that MO is the prime cause of the creation of TOs in engineering firms, which is identified and exploited through the firm's inner processes. From the discussion above, it is proposed that TO, MO, and CE are interlinked. Corporate entrepreneurs in engineering firms are always in search of TO, which comes from MO. Till present, the mediating role of TO on the association between MO and CE has not been tested empirically. Therefore, the present study proposes that:

#### H4:

TO mediates the relationship between MO and CE.

### The moderating role of creative destruction

Schumpeter (1934) suggests that it is the producer who initiates an economic change resulting education of consumers towards the adaptation of new things. The present study proposes that CD intensifies the relationship between MO and TO as it plays a significant role in developing new opportunities. Opportunities usually come from the needs of customers. MO is an approach to business that identifies the customer's needs and tries to develop the products according to their needs. It also becomes a means of the CD because consumers can provide feedback about products, which provides a TO to the corporate entrepreneurs in product improvement. Thus, we hypothesised that:

#### H5:

CD intensifies the relationship between MO and TO.

**Fig. 2:**
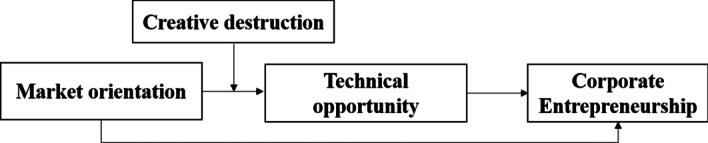
Proposed model

The proposed research model (Fig. 2) from the above-hypothesised relationships is as under:

## Methods

Data collection has been done from engineers in engineering or managerial roles working in private firms. Data collection has been done using convenience sampling to approach engineers from industrial sectors operating in different geographical areas of Pakistan. In ENTR research, this type of non-probability sampling has often been used despite generalisability concerns (Alam et al., 2020; Munir et al., 2019). Online questionnaires link was sent to respondents after acquiring their consent to participate in the study. One hundred thirty-two responses were included in the study. Responses of 81 male engineers (61%) and 51 female engineers (39%) form part of the study for analysis. 42 (32%) engineers who form part of the study are from the textile industry, 29 (22%) from automotive manufacturing, 36 (27%) from power sector maintenance, and 26 (19%) from the telecom engineering sector.

MO and CE measurement scales have been adapted from existing literature as these latent constructs are well established in the literature. Scale for MO has been adapted from Saraf et al., (2007). Sample items include "*our philosophy of doing business is driven by the need of putting customers first*". Hornsby et al. (2002) have developed a comprehensive scale to measure CE that has been validated by Rutherford and Holt (2007). The 48 items Corporate Entrepreneurship Assessment Instrument (CEAI) was used to measure five dimensions of CE as a second-order construct. CD as a concept has been specified by Schumpeter (1934); however, researchers have not used it as a construct to test entrepreneurial phenomena or processes. Scale for CD was developed for this study. Items of the scale include (1) new products replace the existing products in markets; (2) changing environment creates the need for new technologies and products; (3) many existing products in market become obsolete due to improved new products, and (4) creation of a products or service leads to the replacement of old ones. TO recognition construct is perceived as an equivalent construct to entrepreneurial opportunity recognition. Scale for TO recognition has been adopted from Park et al. (2017). A 5-point Likert scale has been used to measure items of constructs.

Since constructs used in the model are latent constructs with multiple measurement items, the multivariate technique SEM is most appropriate for this study. SEM is a widely used approach (Alam et al., 2019; Alam et al., 2020; Alam et al., 2020) in management science and ENTR-based studies (Sarstedt et al., 2014). Covariance-based and PLS are the two primary methods in SEM (Hair et al., 2019). In this study, PLS-SEM is used for data analysis as the research model is unique, and the sole purpose is to investigate relationships at the theoretical level (Hair et al., 2019). PLS-SEM is appropriate due to small sample size requirements, level of measurement, friendly interface, and normality of data issues (Chin & Newsted, 1999).

Through self-report scales, the data collection for independent and dependent variables was done simultaneously, which could raise the issue of common method bias in study results. In line with the recommendations of Kock (2015), variation inflation factor (VIF) of constructs was obtained to observe pathological collinearity, which could be used as an indication of contamination of data due to common method bias. The factor-level VIF values of constructs from the full collinearity test were obtained, and all values were found lower than the threshold of 3.3. Hence, the model is free of common method bias (Kock, 2015, p. 7).

## Results

### Measurement model

The study employed confirmatory factor analysis (CFA) for first and second-order constructs to verify the interrelatedness of constructs in the hypothesised model. The validity of the reflective measurement model was determined by examining its internal consistency and the convergent and discriminate validities. The reliability of all reflective measures was computed by using composite reliability (CR) and Cronbach's α value. Values for each variable satisfied the recommended threshold level, which was above 0.7 (Fornell & Larcker, 1981). Average variance explained (AVE) has been used to compute convergent validity. Items included to measure constructs have loadings above the threshold value of 0.7 (Hair et al., 2019). The measurement model assessment results for first- and second-order constructs comprising indicator reliability, internal consistency reliability, and convergent validity are presented in Table 1.

**Table 1:** Assessment of measurement model for first- and second-order constructs

Latent variables	Dimension	Items	Indicator reliability		Internal consistency reliability		Convergent validity
			First-order loadings	Second-order loadings	α	CR	AVE
MO	–	MO_1	0.858	–	0.882	0.926	0.806
		MO_2	0.91				
		MO_3	0.924				
CD	–	CD_1	0.879	–	0.88	0.914	0.727
		CD_2	0.925				
		CD_3	0.884				
		CD_4	0.707				
TO	Opportunity discovery (OD)	OD_1	0.755	0.917	0.896	0.923	0.707
		OD_2	0.88				
		OD_3	0.902				
		OD_4	0.795				
		OD_5	0.862				
	Opportunity creation (OC)	OC_1	0.795	0.726	0.838	0.881	0.554
		OC_2	0.791				
		OC_3	0.693				
		OC_4	0.774				
		OC_5	0.757				
		OC_6	0.645				
CE	Management support (MS)	MS_1	0.811	0.843	0.935	0.949	0.756
		MS_2	0.896				
		MS_3	0.845				
		MS_4	0.911				
		MS_5	0.895				
		MS_6	0.856				
	Work discretion (WD)	WD_1	0.765	0.86	0.784	0.85	0.537
		WD_2	0.792				
		WD_3	0.768				
		WD_4	0.0784				
		WD_5	0.718				
	Rewards/reinforcement (RR)	RR_1	0.845	0.911	0.859	0.9	0.694
		RR_2	0.902				
		RR_3	0.742				
		RR_4	0.834				
	Time availability (TA)	TA_1	0.727	0.742	0.76	0.849	0.596
		TA_2	0.838				
		TA_3	0.815				
		TA_4	0.669				
	Organisational boundaries (OB)	OB_1	0.891	0.903	0.927	0.946	0.815
		OB_2	0.929				
		OB_3	0.894				
		OB_4	0.897				

The heterotrait–monotrait ratio of correlations (HTMT) has been used to assess discriminant validity (threshold < 0.85) of first and second-order constructs (Henseler et al., 2015). The discriminant validity results for first-order constructs and second-order constructs are presented in Table 2 and Table 3. Multi-collinearity issues were not there in data since VIFs were found less than 5 (Hair et al., 2019). The measurement model was found to be appropriate for the assessment of the structural model and further analysis.

**Table 2:** Discriminant validity (HTMT) for first-order constructs

	CD	MO	MS	OB	OC	OD	RR	TA	WD
CD									
MO	0.823								
Management support (MS)	0.081	0.053							
Organisational boundaries (OB)	0.379	0.374	0.07						
Opportunity creation (OC)	0.102	0.057	0.703	0.091					
Organisational discovery (OD)	0.416	0.397	0.08	0.547	0.092				
Rewards/reinforcement (RR)	0.541	0.527	0.084	0.653	0.155	0.752			
Time availability (TA)	0.127	0.117	0.533	0.069	0.69	0.144	0.121		
Work discretion (WD)	0.181	0.141	0.137	0.163	0.172	0.116	0.133	0.142

**Table 3:** Discriminant validity (HTMT) after generating second-order constructs

	CE	CD	MO	TO
CE				
CD	0.473			
MO	0.436	0.816		
TO	0.795	0.692	0.646	

### Structural model

The proposed hypotheses (H1–H5) were tested by assessing the structural model for higher-order constructs. Hypotheses testing has been done through examination of the significance of the relationships among constructs with path coefficients. For assessing the significance of path coefficients, the bootstrap resampling method in PLS has been performed (Chin, 1998).

Results indicate that MO is positively associated with CE (*β* = 0.19, *p* < 0.05), supporting H1, TO is positively associated with CE (*β* = 0.202, *p* < 0.05), supporting H2 and MO is positively associated with TO (*β* = 0.289, *p* < 0.05), supporting H3. As Shrout and Bolger (2002) proposed, the bootstrap procedure was performed to verify the significance of indirect effects on the 5000 samples. This procedure provides a point estimate of the indirect effect at the 95% confidence interval (CI) through bootstrap approximation (MacKinnon et al., 2004). The indirect effect of MO on CE through TO (estimate = 0.059, 95% CI = [0.015, 0.082], *p* < 0.05) was significant as the confidence intervals did not include zero.

**Fig. 3:**
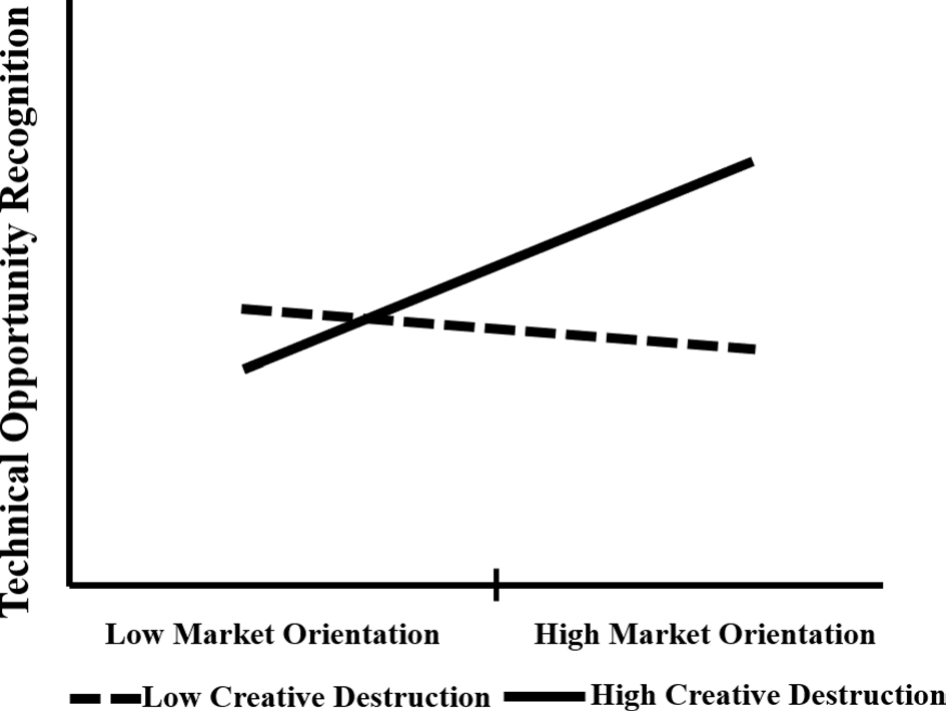
Moderating effect of CD on MO and TO

CD significantly moderates between MO and TO (estimate = 0.141, 95% CI = [0.026, 0.337], *p* < 0.05). Figure 3 shows the nature of the moderating effect obtained through plotting values of unstandardised β of moderating effect (CD), independent (MO) and dependent variables (TO). The plot shows, with a low level of CD, MO has less impact on TO. However, with the high level of CD, MO has a high impact on TO, hence, CD strengthens the positive effect of MO on TO.

## Discussion and future research directions

This study has suggested a model incorporating Schumpeterian opportunity creation and CD concept to explain the emergence of CE. Most CE research is limited to seeking antecedents in various contexts (Alam et al., 2020; Alam et al., 2020; Woo, 2018), as the phenomenon is still in the exploratory stage. This is perhaps due to various synonymous terms explaining similar phenomena like corporate venturing, strategic renewal, and intrapreneurship (Zahra et al., 1999). Also, the dependent variable issue in CE research restricts researchers from looking for practical outcomes, which could be explained through a sound theoretical basis.

Results suggested a positive MO relationship with CE in line with previous research (Barrett et al., 2012). Although previous research on the relationship between MO and CE was carried out in various contexts (Ahmed, 2016), this relationship merits focus specific to engineering or technology firms.

The findings of this study confirmed that MO and TO are essential antecedents of CE in engineering firms. The TO recognition is somewhat considered equivalent to the entrepreneurial opportunity that required deliberation by academic researchers. This study suggests that the Schumpeterian concept of CD explains TO in engineering firms, and theoretical perspectives that have been investigated in other contexts need further empirical evaluation. Schumpeter (1934) and Shane (2003) viewed that technological development occurs well in suitable environmental conditions, inspiring individuals to seek opportunities. Additionally, findings also extend Lee and Venkataraman (2006) argument about the limitations of existing literature towards the importance of external context in opportunity recognition. Lee and Venkataraman (2006) assert that existing literature has emphasised more on individual factors that need to be considered along with external factors in the opportunity recognition process. The present study has considered only the external factors in the opportunity recognition process. Future studies could compare the individual and external factors in other contexts to investigate which (individual or external) explains opportunity recognition better. Previous research supports this study's findings on opportunity and CE results (Pech & Cameron, 2006), and innovation and venture growth can be explained as a function of opportunity recognition (Sambasivan et al., 2009).

Although this study has investigated external factors in opportunity recognition to impact CE, opportunity recognition is a vast phenomenon, and MO is just one dimension. Future studies could consider established antecedents of opportunity recognition (nations' cultural and social characteristics) in similar contextual studies.

The results of this study support the intensifying CD impact on MO and TO link in the proposed model. CD's impact on the proposed relationship provides empirical backing to Schumpeter's idea. Although Schumpeter's work has great significance in ENTR literature, few researchers have identified limitations to his work on understanding ENTR. Regardless of the famous phrase "CD", Schumpeter's work explains only the proceeding of novelty, but fails to explain entirely new entrepreneurial creativity as his overall framework favours human will against the subjectivism of the human mind (Witt & Foster, 1992). Schumpeter supports disruption of economic equilibrium by entrepreneurs to attain another one is the very idea of CD, which is contrary to Kirzner's (1997) approach of opportunity recognition and market equilibrium.

The proposed model contributes to the ENTR literature in general and CE literature, specifically, in several ways. First, this model proposed another view to classical debate on the opportunity and distinguished entrepreneurial opportunity from TO in line with Schumpeterian argument on technical innovation through invention. The debate on whether CD creates market disequilibrium or equilibrium is the result of CD, which future researchers can view to explore the CD phenomenon in depth. Also, whether CD as a phenomenon is useful in the overall economic fabric from the capitalist or socialist point of view can be further examined. The proposed model adds elements to the concept and facilitates understanding of CE from opportunity and market antecedents.

Most innovative technological products result from technological breakthroughs in the field with a somewhat vague idea of its market acceptability (Bennett & Cooper, 1979, p. 77). A technical breakthrough in any field is not necessarily bound to MO as market needs and varied customer demands could fail an otherwise quality product (Kerby, 1972) because customers have their perceptions and needs about familiar things (Bennett & Cooper, 1981). Also, only customer orientation will not be enough as getting closer to customers can also hinder innovation rather than its promotion (MacDonald, 1995). Hence, if TO emanates from market demand (or MO), it can initiate CE activity in engineering firms that could contribute to the economy. MO of products is linked with the overall status of existing products about whether current products match customer's expectations? CD will fill gaps where current market products are getting obsolete due to new trends and emerging demands. This will stimulate TO recognition, and the results of this study empirically prove this vital link for incorporation in existing theories to explain CE antecedents.

## Conclusion

In conclusion, CE distinctions in engineering firms are to be taken from the Schumpeterian idea of CD, which seems less relevant in less technically oriented industries. TO recognition is the main driving force, which should emanate from MO for a successful CE process in overall benefit for firms and the economy. The overall philosophy of looking at future market needs and customer demands requires a breakthrough in the current century in line with the one proposed by Schumpeter in the last century. As of today, the Schumpeterian idea seems more relevant to opportunity recognition. Ideas are like products that must obsolete with time to move ahead. There seems space for arguments, ideas, and philosophies that could bring more relevant products and services to the market by exploiting the gaps timely due to the natural obsolescence of products and services.

## Data Availability

The datasets used and/or analysed during the current study are available from the corresponding author on reasonable request.

## Abbreviations

CD: Creative destruction
CE: Corporate Entrepreneurship
TO: Technical opportunity
MO: Market orientation
SEM: Structural Equation Modelling
PLS: Partial Least Square
